# Rapid Bacterial Species Delineation Based on Parameters Derived From Genome Numerical Representations

**DOI:** 10.1016/j.csbj.2018.12.006

**Published:** 2019-01-09

**Authors:** Denisa Maderankova, Robin Jugas, Karel Sedlar, Martin Vitek, Helena Skutkova

**Affiliations:** Department of Biomedical Engineering, Faculty of Electrical Engineering and Communication, Brno University of Technology, Technicka 12, 61600 Brno, Czech Republic

**Keywords:** Bacterial genome, Species delineation, Comparative genomics, Numerical representation, Genomic signal processing

## Abstract

Species delineation based on bacterial genomes is an essential part of the research of prokaryotes. In silico genome-to-genome comparison methods are computationally demanding, but much less tedious and error prone than the wet-lab methods. In this paper, we present a novel method for the delineation of bacterial genomes based on genomic signal processing. The proposed method uses numerical representations of whole bacterial genomes, phase signal and cumulated phase signal, from which four parameters are derived for each genome. The parameters characterize a genome and their calculation is independent of the other genomes comprising a delineation dataset. The delineation itself is processed as a calculation of the parameters' average similarity. The method was statistically verified on 1826 bacterial genomes. A similarity threshold of 96% was set based on the receiver operating characteristic curve that featured sensitivity of 99.78% and specificity of 97.25%. Additionally, comparative analysis on another 33 bacterial genomes was conducted using standard delineation tools as these tools were not able to process the dataset of 1826 genomes using desktop computer. The proposed method achieved comparable or better delineation results in comparison with the standard tools. Besides the excellent delineation results, another great advantage of the method is its small computational demands, which enables the delineation of thousands of genomes on a desktop computer. The calculation of the parameters takes tens of minutes for thousands of genomes. Moreover, they can be calculated in advance by creating a database, meaning the delineation itself is then completed in a matter of seconds.

## 1 Introduction

One of the first tasks in the research of any newly studied organism lies in its correct taxonomic placement. While the taxonomy of higher eukaryotes is less complicated for relatively easily distinguishable species formed by a group of organisms that can interbreed [1], the majority of the Tree of Life is formed by microbial species. This domination lies in their abundance, with the estimation of the total number of microbial cells on Earth thought to be 10^30^, and in their richness as this amount is formed mainly by 10^6^–10^8^ separate prokaryotic genospecies [2].

Unfortunately, compiling the taxonomy of asexual microbial organisms is not easy and requires a combination of genotypic, phenotypic, and chemotaxonomic information [3]. Due to the advances in biological molecular techniques, the genotypic traits play the main role in microbial species delineation. While some of the genotypic techniques utilize selected barcoding of parts of genomes, the others compare whole genomes. The first group mainly uses techniques for massive genotyping of pathogenic bacteria in epidemiologic studies and includes the utilization of electrophoresis, PCR, and amplicon sequencing [[4], [5], [6], [7]]. The second group serves as a tool for the correct taxonomic placement of a new organism.

In the past, only a few techniques could offer genome-wide comparisons between organisms and DNA–DNA hybridization was considered the recommended standard for delineating species for a long time [8]. A massive reduction in sequencing costs brought a new, wide range of bioinformatics strategies for species delineation in silico by comparing their genome sequences. These included a wide range of techniques for calculating average nucleotide identity (ANI) [9,10]. The ANI of two genomes was first calculated using all shared orthologous protein coding genes [9] and the method was later improved by cutting one genome into 1020 bp fragments that are searched for in the second genome using the BLAST algorithm [11].

Another approach to calculating ANI searches for maximal unique matches (MUM) based on alignment via suffix trees [12,13]. These computationally derived similarities are closely related to former lab-derived hybridization values [14] and can be easily obtained from genome sequences. The taxonomic placement of every new genome should be verified using these approaches as many of the genomes in the databases are mislabeled [15]. For this purpose, there is a wide range of online and standalone tools, e.g. JSpeciesWS [16], Orthologous Average Nucleotide Identity Tool (OAT) [17], Genome-to-Genome Distance Calculator (GGDC) [18], ANI tool by Kostas lab [14], ANItools web [19], dRep [20], Microbial Species Identifier (MiSI) [21], etc.

For the purpose of species delineation using ANI-based methods, a query genome is compared with many other genomes in a database. This task is computationally very demanding as the query genome has a very low similarity to most of the compared genomes that belong to different taxonomic groups. Most of the comparisons are unnecessary and massively increase the computational time.

In this paper, we present an alternative approach for species delineation utilizing four statistical parameters derived from the original genome using genomic signal processing. We used phase signals and cumulated phase signals [22], which are suitable for pairwise and multiple comparisons [23,24]. From these signals, we were able to derive four unique values characterizing individual genomes, which led to a massive reduction of data without affecting the results of the comparison for delineation purposes [25]. A calculation of the similarity of these parameters expresses the similarity between the genomes. Moreover, our method for species delineation significantly reduces the necessary computational time and the delineation accuracy is better or at least comparable with the accuracy of the other tools.

## 2 Methods

### 2.1 Statistical Parameters Representing Whole Genome

There are many types of numerical representations of nucleotide sequences [[26], [27], [28]]. Each numerical representation highlights the different characteristics of an original nucleotide sequence and is suitable for a different type of subsequent analysis. We chose to use the phase signal and the cumulated phase signal, which are very easy to calculate and represent the DNA sequence with a vector of numerals instead of symbols. The phase signal is a sequence of values corresponding to the phases of complex numbers assigned to each nucleotide. The assignment is not arbitrary and it is a projection of the nucleotide tetrahedron to the complex plane, where all nucleotide IUPAC symbols can be represented [22]. The numerical map is: A = +1 + j, C = −1 – j, G = −1 + j, T = +1 – j, R = +j, Y = −j, S = −1, W = +1, M = K = *N* = 0. The respective phases' values in radians are: A = *π*/4, C = −3*π*/4, G = 3*π*/4, T = −*π*/4, R = *π*/2, Y = −*π*/2, S = *π*, W = 0 or 2*π*, M = K = N = 0.

The cumulated phase is a cumulative sum of all previous phase values, but it can also be calculated directly from the DNA sequence according to Eq. 1 [22]:

(1) $$c_{k}=\frac{\pi }{4}\left[3\left(n_{G,k}-n_{C,k}\right)+\left(n_{A,k}-n_{T,k}\right)\right],$$

where *n*_*A,k*_, *n*_*C,k*_, *n*_*G,k*_, and *n*_*T,k*_ refer to several corresponding nucleotides from the beginning of the sequence to the position *k*. The phase signal and the cumulated phase signal have the same length *L* as the original sequence and a reverse transformation is possible. An advantage of the cumulated phase signal is its suitability for the visualization of the whole genome sequence. The visualization can reveal the global trend of the genome, which is not noticeable in the original symbolic sequence or phase signal [28]. For example, the majority of bacterial genomes have a characteristic arrow shape when the cumulated phase signal begins in the region of replication origin (oriC). This helps to predict the position of the oriC region in newly assembled genomes [29].

For a whole genome comparison, it is preferable that sequence records start in the same position and the oriC region is an obvious choice. Unfortunately, genomes starting in another position may still occur in the GenBank database. To eliminate the possible influence of different starts, a simple signal-based rearrangement of sequences was made in a similar manner as was used for the purpose of oriC localization [29].

**Fig. 1:**
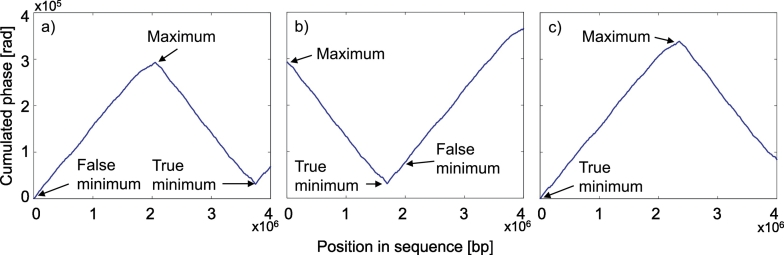
Three-step rearrangement of the cumulated phase signal of the whole bacterial genome of *Bordetella parapertussis*: a) the genome does not begin in the oriC region and the cumulated phase has a false minimum; b) rearrangement according to the global maximum; c) rearrangement according to the true minimum.

We used a very simple and computationally undemanding three-step method that only required the identification of the global maximum and minimum. Firstly, a cumulated phase signal of the sequence was calculated. The genome record may begin in an arbitrary region and thus, the first value of the cumulated phase signal may be a false minimum. Secondly, the maximal value of the cumulated phase signal was found, and the signal was rearranged to begin at the position of the maximal value. This rearrangement means that a part of the signal from the beginning to the maximum was simply moved behind the last signal's value and the last signal's value was added to each value of the moved part to make an offset (see Fig. 1). The symbolic sequence was rearranged accordingly. The last step was a localization of the true minimal value, which was the global minimum of the rearranged signal. The symbolic sequence and the cumulated phase signal were again rearranged to begin at the position of the true minimum. The value of the true minimum was then subtracted from all the signals' values so the cumulated phase signal's first value was 0. Subsequently, the phase signal was calculated from the rearranged sequence.

Many diverse parameters representing global features of individual genomes can be calculated from these two signals. We tested common statistical and mathematical parameters, such as the standard deviation, the sum of differences of adjacent signal values, the length of signal, the area under the cumulated phase signal, different types of angles in the cumulated phase signal, distribution of phase signal values, etc. Based on cross-correlation analysis and analysis of the parameter's value distribution for genomes of the same species and different species, the number of final parameters was reduced. Four parameters were chosen as suitable representatives of genome variability. Together, they exhibited excellent discriminative power.

The first parameter was a sum of the differences of the adjacent phase signal values divided by the length of the signal according to:

(2) $${\mathrm{Diff}}_{p}=\frac{1}{L}\sum_{k=1}^{L-1}\left|p_{k}-p_{k+1}\right|,$$

where *L* is the length of the signal/sequence and *p*_*k*_ is the phase value at position *k*. The difference depends on an order of nucleotides, e.g. its value for the sequence AAAGGG is 0.26 and for the sequence AGAGAG, which has the same nucleotide content but in a different order, it is 1.31.

The second parameter *Tr*_*0*_ was the number of transitions between the positive and negative values of the phase signal and vice versa. The count *Tr*_*0*_ corresponds to the sum of dinucleotides AC, AT, CA, CG, GC, GT, TA, and TG that occurred in the symbolic sequence. The third parameter *Tr*_*CG*_ was the number of all possible transitions between the phase signal's values corresponding to the nucleotides C and G. *Tr*_*CG*_ is an equivalent of the sum of dinucleotides CC, CG, GC, and GG. Both parameters were normalized by the signal's length *L*–1, which represented the total number of transitions between the two values in the signal. Although these parameters could be calculated directly from the original symbolic sequence, this would require separate calculations for each dinucleotide. The processing of the phase signal required only a few numerical operations (subtractions and sums) applied on the whole signal to obtain counts for all dinucleotides, e.g. sequence CAGGCAG has *Tr*_*0*_ = 3/6 and *Tr*_*CG*_ = 2/6.

The fourth parameter was the average growth angle *A*_*cp*_ of the cumulated phase signal. The angle was calculated as an average value of the angles for *N* positions from the beginning of the signal to the maximal value of the signal:

(3) $$A_{\mathrm{cp}}=\frac{1}{N}\sum_{k=1}^{N}A_{\mathrm{cp}}^{k},\;A_{\mathrm{cp}}^{k}=\tan^{-1}\left(\frac{c_{\mathrm{ki}}}{i}\right),\;i=k\frac{i_{\max}}{N},$$

where *c*_*ki*_ is the cumulated phase value at position *ki* and *i*_*max*_ is the position of the maximal value of the cumulated phase signal. The number of positions was set to *N* = 10 as a trade-off between precision and computational demands. The cumulated phase signal can have an uneven rise with several local maxima and the angle can vary depending on the position. The average value smooths the local differences of the signal's growth.

**Fig. 2:**
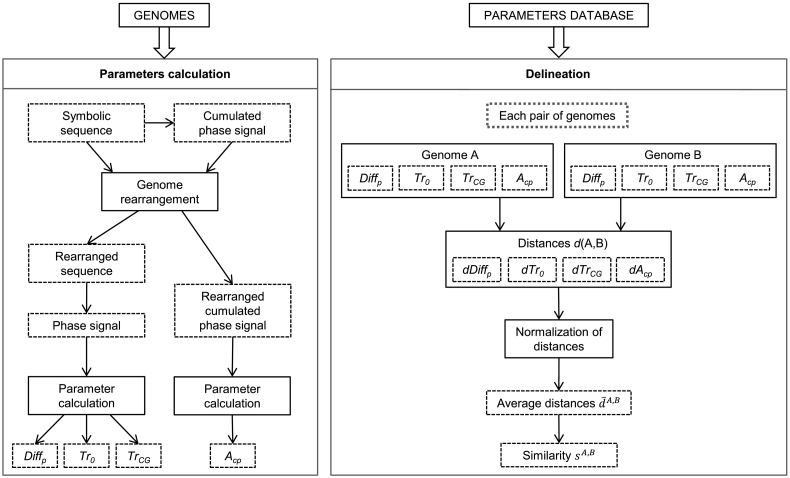
Diagram of the WGP method. Left: independent parameter calculation for genomes; right: subsequent delineation of a dataset.

We propose a rapid delineation method based on these parameters. The Whole Genome Parameter (WGP) method is based on comparing the parameters (*Diff*_*p*_, *Tr*_*0*_, *Tr*_*CG*_, and *A*_*cp*_) of individual genomes. This method does not require any alignment of the genomes or their annotation. Moreover, the parameters can be calculated in advance and saved for further analysis using different compositions of the dataset. Fig. 2 shows the diagram of the WGP method. When processing the delineation analysis of the given dataset, the parameters of all pairs of genomes were compared and the absolute values of the differences between the parameters were calculated. The differences of each parameter were then normalized according to their range in the whole dataset to obtain values from 0 to 1. The normalization was needed to obtain comparable ranges of differences of all parameters. The distance (difference) ${\bar{d}}^{A,B}$ of each pair of genomes *A* and *B* was an average value of the normalized distances calculated for the parameters. A percentage similarity of the genomes was then $s^{A,B}=100*\left(1-{\bar{d}}^{A,B}\right)$.

### 2.2 Standard Delineation Tools

Four freely available web-based and standalone tools that are standardly used for delineation were chosen for testing: JSpeciesWS [16,30], OAT [17], GGDC [18,31], and ANI/AAI-Matrix from Kostas lab [14]. These tools were chosen as they enable delineation analysis using standard desktop computer with Windows operating system which is the most common equipment.

The JSpeciesWS is an online service that provides pairwise comparison of complete or draft genomes by calculating the ANI values or the tetranucleotide signature frequency correlation coefficients (TETRA) [32]. The ANI values are calculated using the BLAST algorithm (ANIb) or the MUMmer alignment tool (ANIm). For both methods, a similarity threshold of 95–96% is suggested to sufficiently differentiate species. The TETRA is an alignment-free method based on characteristic frequency occurrences of all 256 possible combinations of tetranucleotides. Closely related genomes have a similar distribution of the frequencies with a high correlation coefficient of >0.99, which corresponds to the ANI value of >96% [30].

The OAT (Orthologous Average Nucleotide Identity Tool) is standalone tool based on OrthoANI method [17] which is a reciprocal version of ANI calculation using BLAST. The reciprocal means that comparison of two genomes is the same for both pair combinations. Therefore, only half of the comparisons is needed to analyze whole dataset. The similarity threshold is 95–96%.

The Genome-to-Genome Distance Calculator (GGDC) is a web tool that uses statistical models of digital DNA-DNA hybridization and is based on the Genome Blast Distance Phylogeny program (GBDP). First, the BLAST algorithm is applied to find the high-scoring segment pairs (HSPs) between two compared genomes. Second, the GBDP uses three different formulas to calculate the distance between the genomes: sum of all HSPs' lengths/sum of genomes' lengths (ANI-f1), identities in HSPs/sum of all HSPs' lengths (ANI-f2), and identities in HSPs/sum of both genomes' lengths (ANI-f3). Then, the distance is converted to an analogous DNA-DNA hybridization (dDDH) value using a generalized linear model. The formula ANI-f2 is recommended and the threshold for the species delineation is set to 70%.

The ANI/AAI-Matrix is a web tool by Kostas lab. Beside the ANI values, the tool also calculates average amino acid identity (AAI), which is better for less related organisms with ANI <75% [33]. The ANI values are calculated using BLAST and the delineation threshold is 95%.

## 3 Results and Discussion

### 3.1 Statistical Validation of WGP Method

The WGP method was statistically validated on an extensive dataset of whole bacterial genomes that were downloaded from the GenBank database. The dataset comprises 1826 genomes in total. The composition of the dataset was designed to contain enough sequences of the same species and also different species. Within the dataset, the first 350 genomes belong to seven species: *Acinetobacter baumannii*, *Bacillus cereus*, *Bacillus subtilis*, *Escherichia coli*, *Klebsiella pneumoniae*, *Salmonella enterica*, and *Staphylococcus aureus*, where each species is equally represented by 50 genomes.

The remaining 1476 genomes were downloaded from the NCBI Reference Sequence Database. Only one representative genome of each species was included in the dataset, except for the seven species listed above. Within the 1476 genomes, the genomes with synonym species names were not eliminated. This means the dataset may contain more sequences of the same species, except for the first 350 genomes, however, the number is considered low enough that their influence on the analysis was negligible.

**Fig. 3:**
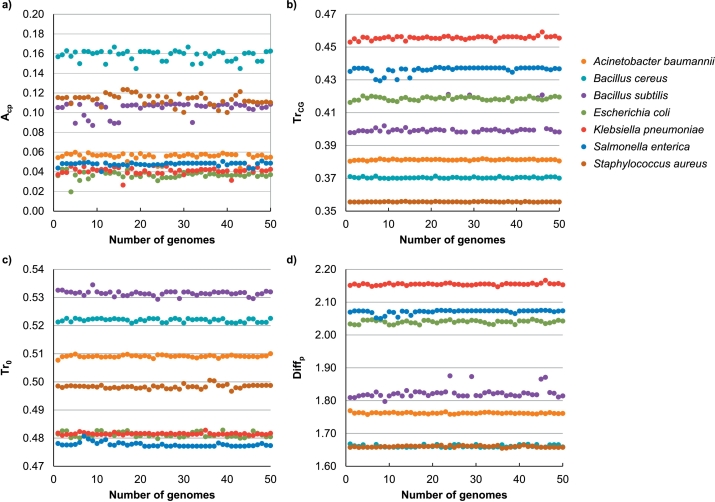
Distribution of the parameters for 350 genomes: a) the average growth angle *A*_*cp*_ of the cumulated phase signal; b) *Tr*_*CG*_ – the number of transitions between the phase values of nucleotides C and G; c) *Tr*_*0*_ – the number of transitions between the positive and the negative values of the phase signal; d) *Diff*_*p*_ – the sum of differences of the adjacent phase values.

The parameters *Diff*_*p*_, *Tr*_*0*_, *Tr*_*CG*_, and *A*_*cp*_ were calculated for all 1826 genomes. Fig. 3 shows the distribution of the parameters for the seven species represented by 50 genomes each. Any one parameter was not able to sufficiently discriminate all species alone, but each parameter was able to discriminate between different groups of species. For example, the strongest parameter *Tr*_*CG*_ discriminated all species quite well, but there were four genomes of *Bacillus subtilis* that overlapped with *E. coli*. Although the parameter *A*_*cp*_ seemed to be the worst at species discrimination in comparison with the other parameters, a subsequent analysis showed its significance.

The normalized distances of the parameters were calculated for all pairs of genomes, which meant 1.66 million genome-to-genome comparisons. The WGP method is reciprocal which means a comparison of genomes *A* and *B* is the same as *B* and *A.* Therefore, only half of all possible genomes' pairs was calculated. The overall distance ${\bar{d}}_{4}$ of each genome pair was calculated as an average value of the normalized distances of the four parameters. To assess the contribution of each parameter to the overall distance, ${\bar{d}}_{2}\;$was calculated as the average distance of parameters *Tr*_*CG*_ and *Diff*_*p*_ and ${\bar{d}}_{3}$ was calculated as the average distance of parameters *Tr*_*CG*_, *Diff*_*p*_, and *Tr*_*0*_. Genome similarities ${\bar{s}}_{2}$, ${\bar{s}}_{3}$, and ${\bar{s}}_{4}$ and similarities for each parameter (*sDiff*_*p*_, *sTr*_*0*_, *sTr*_*CG*_, and *sA*_*cp*_) were derived from the distances.

**Fig. 4:**
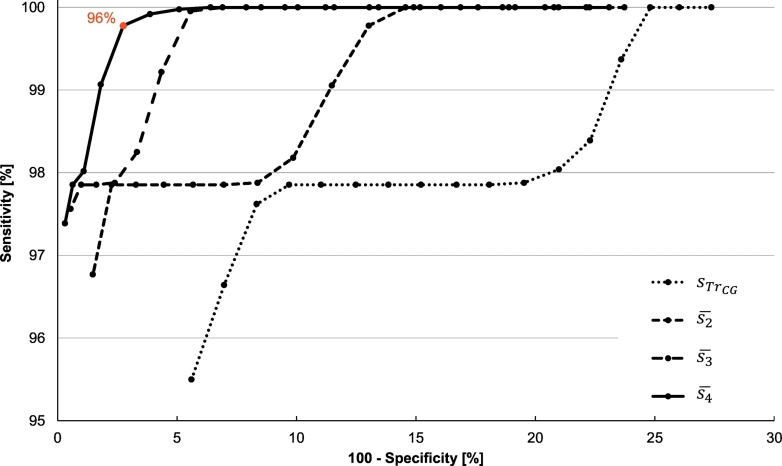
The ROC curves for the delineation based on *sTr*_*CG*_, ${\bar{s}}_{2}$ (the average similarity of parameters *Tr*_*CG*_ and *Diff*_*p*_), ${\bar{s}}_{3}$(the average similarity of parameters *Tr*_*CG*_, *Diff*_*p*_, and *Tr*_*0*_), and ${\bar{s}}_{4}$ (the average similarity of all parameters) for the similarity threshold 90–98%, step 0.5%. The red value is the similarity threshold for which the best sensitivity and specificity results were obtained. (For interpretation of the references to color in this figure legend, the reader is referred to the web version of this article.)

Sensitivity and specificity were calculated for a similarity threshold within the range 90% to 98% (see Additional file 1). The similarity threshold was used to divide the genome similarity values into four groups: true positives (TP) were similarity values above the threshold of a genome pair belonging to the same species, true negatives (TN) were similarity values below the threshold of a genome pair of two different species, false positives (FP) were similarity values above the threshold of a genome pair of two different species, and false negatives (FN) were similarity values below the threshold of a genome pair belonging to the same species. Receiver operating characteristic (ROC) curves were constructed where sensitivity was a function of 100 – specificity (see Fig. 4).

FN could occur only in the case of the 350 sequences of the seven species as the dataset did not contain two or more sequences for any other species. The validation showed a low level of FN. Thus, the sensitivity of the WGP method is very high for delineation based even on only one parameter. The sensitivity decreased with an increase in the similarity threshold level. For a threshold of 98%, the lowest sensitivity of 84.35% belonged to the parameter *A*_*cp*_. The parameter with the best sensitivity of 99.94% for the same threshold was *Tr*_*0*_. The delineation based on the average of all parameters had sensitivity within the range of 97.39% to 100%.

The specificity of each parameter was lower than the sensitivity and in contrast to the sensitivity; it rose with higher values of the similarity threshold. For a threshold of 90%, the specificity ranged from 43.77 to 77.86% and it rose to 85.42–99.69% for a threshold of 98%. The biggest benefit of the combination of parameters was the significant improvement in the specificity. Fig. 4 shows the ROC curves for the delineation based on the similarity of parameter *Tr*_*CG*_, the average of *Tr*_*CG*_ and *Diff*_*p*_, the average of *Tr*_*CG*_, *Diff*_*p*_, and *Tr*_*0*_, and finally the average of all parameters that gave the best results for both sensitivity and specificity. Based on the ROC diagram, the delineation threshold was set to 96%.

When the described genome rearrangement according to the minimal value of the cumulated phase signal was omitted, the delineation results were negatively affected. The sensitivity of the delineation based on ${\bar{s}}_{4}$ decreased by approximately 3% and the specificity by 1% for the suggested threshold of 96% and the results were not better for the other thresholds (see Additional file 1).

### 3.2 Comparison of the WGP Method with the Standardly Used Tools

The extensive dataset used for the statistical validation of the WGP method could not be analyzed by the standardly used tools based on ANI calculation on a standard desktop computer as these methods are extremely computationally demanding and require computing clusters or grid. For comparison purposes, a small dataset containing only 33 bacterial genomes of nine different species was assembled. Each species was represented by at least two strains. Five species were Gram-positive: *Bacillus cereus*, *Bacillus licheniformis*, *Bacillus subtilis*, *Clostridium acetobutylicum*, and *Clostridium beijerinckii*. Four species were Gram-negative: *Escherichia coli*, *Klebsiella pneumoniae*, *Shigella flexneri*, and *Shigella sonnei*. It has been suggested that the genus *Shigella* should be considered as a subgenus of *E. coli* [34,35], however, we decided to count *Shigella* as a different species and test whether the whole genome-based delineation was able to distinguish these genomes from *E. coli*. Additional file 2 provides the names and GenBank accession numbers of all genomes in the dataset.

**Fig. 5:**
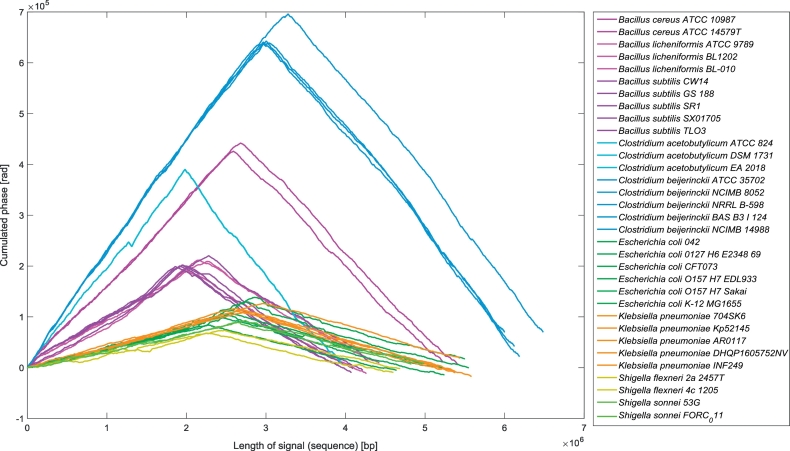
The cumulated phase signals of 33 bacterial genomes of nine different species. Signals of different strains of one species have the same color.

Fig. 5 shows the cumulated phase signals of the 33 bacterial genomes. The Gram-positive bacteria showed significant differences between their signals, whereas the Gram-negative bacteria were not clearly visually separated according to the species. As can be seen, the strains of *Bacillus cereus* differ in length by 187.5 kbp and the other two *Bacillus* species have much shorter genomes. The signal of *B. licheniformis* ATCC 9789 is visually more similar to *B. subtilis* than to the other two *B. licheniformis* strains. Similarly, *B. subtilis* CW14 is closer to the *B. licheniformis* strains. The signals of the three *Clostridium acetobutylicum* strains are visually almost identical and the genomes' lengths are much shorter than the closely related genomes of *Clostridium beijerinckii*, which are the longest genomes in the dataset. One of them, *C. beijerinckii* NCIMB 14988, is from 298.5 kbp to 486.3 kbp longer than the others.

**Fig. 6:**
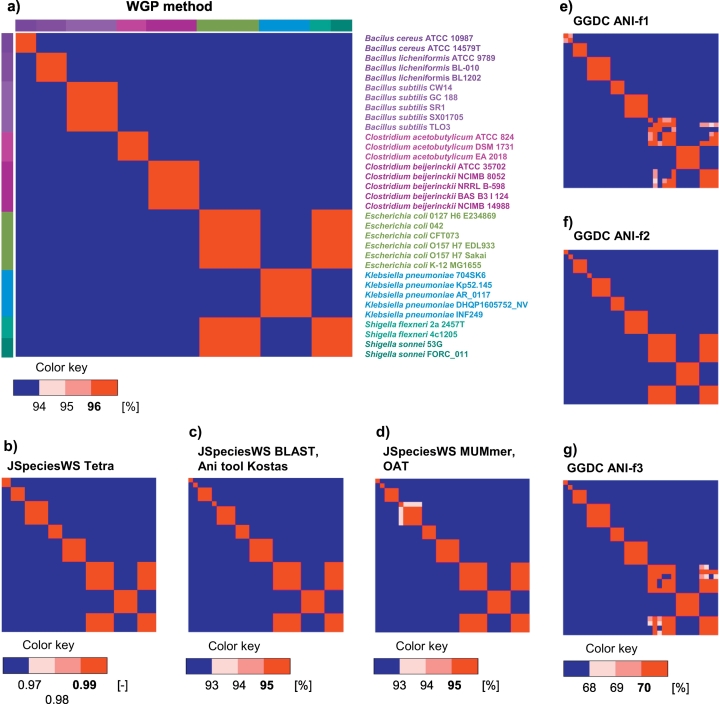
Visualization of the delineation results: a) our proposed WGP method; b) online tool JSpeciesWS using method TETRA; c) online tool JSpeciesWS using BLAST and Ani tool by Kostas lab; d) online tool JSpeciesWS using MUMmer and OAT; e) tool GGDC using formula ANI-f1; f) GGDC tool using the recommended formula ANI-f2; and g) GGDC tool using formula ANI-f3. The methods that have the same heatmap for the given threshold share one subplot. On the diagonal, there is a self-comparison of each genome where self-similarity is 100%. Similarity values above the threshold (bold value in the color key) are highlighted in red and similarity values 2% below the threshold are blue. (For interpretation of the references to color in this figure legend, the reader is referred to the web version of this article.)

All ANI-based tools were used with their default or recommended settings. The delineation results of all the tested methods are visualized as heatmaps (see Additional file 3 and Fig. 6).

The online tool JSpeciesWS can only analyze 15 genomes in one run, therefore, the dataset of 33 genomes needed to be divided into several sub-datasets to achieve all the pairwise comparisons. The tool displays the estimated duration of the computation and for a pair of genomes, the estimated duration was 80 s using BLAST (ANIb), 20 s using MUMmer (ANIm), and 6 s using TETRA. The dataset of 33 genomes required 1056 genome-to-genome comparisons, which had an estimated duration of 23.47 h for ANIb, 5.87 h for ANIm, and 1.76 h for TETRA. For comparison, the computational time for the WGP method was 106 s for the parameter calculations of the 33 genomes and 0.02 s for the delineation itself using a standard desktop computer without parallelization.

The delineation based on the ANIb was unsuccessful for two strains of *Bacillus cereus* with an average similarity 91.05%, which was below the 96% threshold (see Fig. 5c). Likewise, the method had a problem with the *Bacillus subtilis* CW14, which had an average similarity of 92.68% with the other *B. subtilis* strains. An average interspecies similarity of the *Bacillus* species was 69.52%. All strains of the two *Shigella* species had a similarity with *E. coli* above the threshold and the average similarity of this group was 97.26%. All other genomes were correctly delineated. The results based on the ANIm algorithm resembled the ANIb-based results (the similarities differed in tenths of a percent, see Fig. 6d).

The TETRA method of the JSpeciesWS tool correctly delineated the problematic *Bacillus cereus* and *Bacillus subtilis* strains and the *Shigella* strains were again delineated together with the *E. coli* strains (see Fig. 6b).

The OAT in its graphical user interface version accepts up to 10 genomes. It enables to use different BLAST versions and use multiple processing cores on your computer. The 33 genomes dataset was divided into several sub-datasets. The average processing time of one genome pair was 122 s using default BLAST version and running on a desktop computer (Intel ® Core™ i5–3330 CPU @ 3.00GHz 3.20 GHz, 32 GB RAM, 64-bit operational system Windows 7 Professional). Similarly to JSpeciesWS, the delineation was unsuccessful for the two *Bacillus cereus* strains with similarity 91.61% and for *Bacillus subtilis* CW14 strain with average similarity 93.02% in comparison with other *B. subtilis* strains. The similarity values produced by OAT and JSpeciesWS varied by a maximum of several tenths of a percent.

The GGDC web tool analyzed the whole dataset in one run (the limit is 50 genomes). The estimated time for one genome pair was about 1 min, which was approximately 17.6 h for all comparisons of the 33 genomes. The results for the three formulas differed, but the variance was not significant for most of the genomes (see Fig. 6e, f, and g). The results differed significantly only for the two strains of *Bacillus cereus*. In this case, the ANI values of the three formulas were 69.4%, 44.9%, and 65.1%. The lowest ANI value was obtained for the recommended formula ANI-f2 and the value was far below the 70% threshold, whereas the other two values were only slightly under the threshold.

The ANI-f1 values were under the threshold for some *E. coli* strains and for most of the comparisons between the *E. coli* and *Shigella* strains. The comparisons between *Shigella flexneri* and *Shigella sonnei* were above the threshold. Beside *Bacillus cereus*, the ANI-f2 values were also significantly under the threshold for the *Bacillus subtilis* CW14 strain. All *E. coli* strains were above the threshold and the comparisons of *E. coli* with the *Shigella* strains were also above the threshold. The ANI-f3 values were slightly under the 70% threshold for some comparisons between the *E. coli* strains and for the comparisons between *E. coli* and the *Shigella* strains.

Similar to JSpeciesWS, the ANI tool by Kostas lab is based on BLAST and is computationally demanding. The tool limits the analysis to 50 genomes. The delineation results (see Fig. 6c) were under the 95% threshold for the *Bacillus cereus* strains and *Bacillus subtilis* CW14. The *Shigella* strains were delineated together with the *E. coli* strains.

The whole set of 33 genomes was analyzed also by proposed WGP method. The phase signals and the cumulated phase signals were calculated. From the signals, the vector of the four WGP method parameters was calculated for each genome. Each parameter vector was compared with the parameter vectors of all other genomes and the average similarities $\bar{s_{4}}$ were calculated. According to the 96% delineation threshold, all genomes belonging to the same species had a similarity above the threshold (see Fig. 6a). The similarities between genomes of different species were under 94%, apart from the *Shigella* species. *Shigella flexneri* and *Shigella sonnei* were delineated to *E. coli* with an average similarity of 97.84%, whereas the average intraspecies similarity of the *E. coli* strains was 98.51%.

Although another Gram-negative bacteria, *Klebsiella pneumoniae* (average intraspecies similarity 98.80%), had a similar cumulated phase signal to the *E. coli* and *Shigella* species, the average interspecies similarity between *K. pneumonia* and the other Gram-negative bacteria was 85.67%. The highest interspecies similarity of 93.27% occurred between the strains of *Clostridium acetobutylicum* and *Clostridium beijerinckii*. Both species had intraspecies similarities above 99%, even the strain *C. beijerinckii* NCIMB 14988, which had the longest genome. The average similarity between *Bacillus* species was 79.78%. The two strains of *Bacillus cereus*, for which delineation was problematic using the ANI-based methods, had a similarity of 98.90%. The strain *B. subtilis* CW14 had the lowest similarity with other strains of the same species; however, the average value was 96.76%, which was sufficiently above the threshold.

### 3.3 Computational Demands

Table 1 shows the computational time needed for the delineation of one pair of genomes, for the dataset of 33 genomes that was used for the comparative analysis, and for the dataset of 1826 genomes that was used for the WGP method's verification. The WGP method was implemented in Matlab 2015a without parallelization of the task and using a common desktop computer (Intel ® Core™ i5-3330 CPU @ 3.00GHz 3.20 GHz, 32 GB RAM, 64-bit operational system Windows 7 Professional). In Table 1, the WGP method has two values separated by a slash. The first value corresponds to the elapsed time for the parameter calculations and the second value corresponds to the elapsed time for the delineation itself, which means the comparisons of the parameters. For the other tools, the values are based on estimations provided by the tools for one pair of genomes and the dataset of the 33 genomes. The tested tools, using their stand-alone version on the same desktop computer or web version, were not able to process the dataset of 1826 genomes and the time estimations show that such analysis is unrealizable without using large computing clusters or grid. For example, the estimated time for the BLAST-based methods is >3000 days without parallelization of the task. The WGP method calculated the four parameters for the 1826 genomes in 50.8 min and the delineation took 19.9 s. With parallelization using four cores, the times for the WGP method were four times smaller.

**Table 1:** The elapsed and estimated processing times of the WGP method and the standardly used delineation tools.

Method/tool	Time [sec]		
	2 genomes	33 genomes	1826 genomes
WGP	4.5/0.006	71.6/0.013	3048/19.9
JSpeciesWS BLAST, ANI tool by Kostas	*80*	*84,480*	266.6*10^*6*^
JSpeciesWS MUMmer	*20*	*21,120*	66.6*10^*6*^
JSpeciesWS TETRA	*6*	*3168*	9.9*10^*6*^
OAT	*122*	*64,416*	203.3*10^*6*^
GGCD ANI-f1, ANI-f2, ANI-f3	*60*	*63,360*	199.9*10^*6*^

Legend: The elapsed and estimated processing times are for one pair of genomes, the dataset of 33 genomes, and the dataset of 1826 genomes without parallelization of the task. For the WGP method, the first value is the parameter calculation time and the second value is the time taken for the comparison of the parameters. Estimated times are in italics.

The reciprocal methods (WGP, TETRA, and OAT using OrthoANI method) have advantage as they analyze each genome pair once; the similarity does not depend which genome serves as a query. Therefore, the methods compare (*N*x*N*–*N*)/2 genome pairs where *N* is the number of genomes. Non-reciprocal methods compare *N*x*N*–*N* genome pairs. The number of comparisons was reflected in the processing time estimations.

## 4 Conclusions

The proposed WGP method uses four parameters to globally represent a genome. The parameters are calculated from the phase signal and the cumulated phase signal, which are numerical representations of the genome's DNA sequence. The parameters are the sum of the differences of adjacent phase signal values, the number of transitions between the positive and the negative values of the phase signal, the number of transitions between the phase values corresponding to the nucleotides C and G, and the average growth angle of the cumulated phase signal. The parameters' calculation is fast, straightforward, deterministic, and independent of the composition of a dataset. These parameters can be calculated in advance and stored in a database to be used anytime in different delineation analyses. The WGP method enables delineation of an extensive dataset on a standard desktop computer.

The method was verified on a dataset of 1826 RefSeq bacterial genomes. Any WGP method parameter alone was not sufficient to delineate the genomes with sufficiently high sensitivity and specificity. The best results were obtained for an average distance of all four parameters. Based on the verification and the ROC curve, the similarity threshold was set to 96%. For this threshold, the WGP method had a sensitivity of 99.78% and a specificity of 97.25%.

As the standardly used delineation tools were not able to process the dataset of 1826 genomes using desktop computer due to the computational demands and their restrictions on dataset size, the comparison of methods/tools was conducted using a much smaller dataset comprising only 33 bacterial genomes of nine species. The ANI-based methods had a problem with delineating some strains of *Bacilus cereus* and *Bacilus subtilis*. All the compared tools and the WGP method assigned the strains of *E. coli*, *Shigella flexneri*, and *Shigella sonnei* to one group. The analysis showed that these species are difficult to distinguish on a whole-genome level.

Besides the *E. coli* and *Shigella* group, the WGP method produced a perfect delineation faster than the ANI-based methods. Moreover, the ANI-based methods except OrthoANI tool analyze one pair of genomes twice because one genome serves as a “query” and the *sec*ond as a “reference” and the results of the comparisons slightly differ. This double analysis increases the computational time. This is not an issue for the WGP method as the similarity between genomes *A* and *B* is the same as for *B* and *A*, therefore, only half the comparisons are needed. Furthermore, the BLAST and MUMmer algorithms are very complex and computationally demanding, making an analysis of hundreds or even thousands of genomes impossible on a desktop computer and require large computing clusters or grid.

One of the biggest problems in contemporary bacterial genome research is the huge amounts of data that need to be analyzed. The methods used to process these data need to be computationally effective, which is not the case for the available delineation tools. The proposed signal-based method can tackle this problem reliably in what has previously been an unattainable time, even without task parallelization. Additional parameters can be added to the existing ones if needed (by extending the vector of four parameters). For example, to obtain sufficient resolution within species or groups of closely related species.

The WGP method is based on four parameters that globally represent genomes and is a powerful tool for the processing of huge amounts of data. We consider this method to be a newly proven concept that has significant advantages for genomic signal processing. As the development of nanopore sequencing technology is expected in the near future, genomic signal processing methods may be of great importance. The nanopore technology produces a current signal that has to be converted into a symbolic sequence. This conversion can be skipped and the genome can be analyzed in its signal representation using the genomic signal processing methods. The WGP method derives the parameters from the phase representations of a genome and equivalent parameters can be derived directly from the nanopore produced signal.

We have shown that a delineation based on several parameters representing a whole genome is not only possible, but highly effective and precise. The aim of third-generation sequencers is to enable everybody everywhere to perform DNA sequencing that requires only low computational demands. Our delineation method reduces a whole genome from millions of symbols to only four significant values. This enables the comparison of extensive numbers of microorganisms even without online access to large databases.

The WGP method can be downloaded as Matlab source codes: https://www.ubmi.feec.vutbr.cz/en/publications/wgp-genome-delineation/. The present version of the software is not suitable for comparison of genomes assembled in multiple scaffolds.

## Funding

This work was supported in part by the grant project of the Czech Science Foundation [GACR 17-01821S].

## Conflict of Interest

The authors declare that they have no conflict of interest.

## Appendix A Supplementary data

Supplementary material 1 Sensitivity and specificity of the statistical validation of the WGP method. Supplementary material 1

Supplementary material 2 The names and GenBank accession numbers of 33 genomes in the dataset for the comparative delineation analysis. Supplementary material 2

Supplementary material 3 The delineation results for all tested methods/tools for the dataset of 33 genomes. Supplementary material 3
